# Ultrasonographic Features of Gastropathy in Dogs with Acute Kidney Injury and Acute-on-Chronic Kidney Injury

**DOI:** 10.3390/ani15182666

**Published:** 2025-09-11

**Authors:** Caterina Puccinelli, Tina Pelligra, Astrid Bracco, Ilaria Lippi, Francesca Perondi, Tommaso Mannucci, Simonetta Citi

**Affiliations:** Department of Veterinary Sciences, University of Pisa, 56024 Pisa, Italy; caterina.puccinelli@unipi.it (C.P.);

**Keywords:** uremic gastritis, uremic gastropathy, ultrasound, dog, AKI, ACKD

## Abstract

**Simple Summary:**

Dogs with acute kidney injury (AKI) or acute-on-chronic kidney disease (ACKD) often present with gastrointestinal signs, yet the stomach’s ultrasonographic appearance in these conditions has not been previously described. This retrospective study aimed to characterize gastric abnormalities observed on ultrasound in dogs with AKI or ACKD. A total of 113 dogs diagnosed with AKI or ACKD were included, all showing ultrasonographic changes in the stomach. Gastric wall thickening was present in all cases. In 86.7% of dogs, abnormalities involved the mucosal and/or submucosal layers. Mucosal changes alone were found in 54.9% of cases; the most common mucosal alteration was a hyperechoic band. Submucosal changes alone, typically characterized by thickening and decreased echogenicity—suggestive of edema—were found in 20.3% of dogs. Mucosal lesions were more frequent in dogs with ACKD, while submucosal changes were more often seen in AKI. These findings provide the first detailed description of stomach ultrasound features in dogs with AKI and ACKD.

**Abstract:**

Despite the frequent gastrointestinal involvement, no studies have described the ultrasonographic appearance of the stomach in dogs with acute kidney injury (AKI) or acute-on-chronic kidney disease (ACKD). This retrospective study aimed to characterize the ultrasonographic features of gastropathy in such patients. Dogs diagnosed with AKI or ACKD and showing gastric abnormalities on ultrasound between January 2014 and January 2024 were included. A total of 113 dogs met the inclusion criteria. Gastric wall thickening was observed in all cases. Mucosal and/or submucosal abnormalities were present in 86.7% of dogs. Mucosal changes alone were found in 54.9%, including hyperechoic band or stripe and diffusely increased echogenicity, suggestive of possible mucosal mineralization. The most common mucosal finding was a hyperechoic band (48.4%), associated with higher calcium–phosphorus product levels. Submucosal changes alone were seen in 20.3% of dogs, typically as thickening with decreased echogenicity, consistent with possible submucosal edema. Both mucosal and submucosal abnormalities were present in 11.5% of cases. Mucosal changes were more frequent in ACKD, while submucosal alterations were more common in AKI. This study provides the first detailed description of gastric ultrasonographic features in dogs with AKI and ACKD.

## 1. Introduction

Gastritis is a general term referring to a non-specific inflammatory syndrome affecting the stomach, which can occur acutely or chronically [1,2]. In response to a triggering factor, various cell types can release inflammatory and vasoactive mediators that initiate the inflammatory response [3]. This can alter the physiological mucosal barrier, potentially resulting in abnormalities of the microvascular blood flow with secondary hypoxia [3]. Possible consequences include hemorrhage, edema, necrosis, and ulceration of the gastric wall [3].

Uremic gastritis or gastroenteritis is a term commonly used by veterinarians to refer to the range of gastrointestinal symptoms observed in dogs and cats with chronic renal disease [4,5,6,7]. Indeed, gastrointestinal manifestations of uremia frequently occur in these patients [6]. These signs may include vomiting, diarrhea, and gastrointestinal bleeding [6,7,8,9].

Gastrointestinal signs in patients with chronic kidney disease (CKD) have been attributed to higher circulating uremic toxins and decreased renal clearance of gastrin, a hormone that, in excess, can cause gastric hyperacidity and ulceration [7,8,10]. In uremic dogs specifically, progressive uremia allows urea to diffuse from the interstitial fluid into the stomach, resulting in damage to the epithelial cell layer of the gastric wall [1,11].

In humans with end-stage kidney disease, gastrointestinal diseases, such as peptic ulcers and erosive gastroduodenitis, are common [12,13]. On the other hand, in CKD dogs, gastroduodenal ulceration and erosion are not commonly reported [6,14].

In dogs, the term “uremic gastritis or gastropathy” has been characterized histopathologically by features such as mineralization, edema of the lamina propria, atrophy of gastric glands, and vascular injury with thrombosis [6,7,15]. Other histological lesions that may be observed include necrosis of the parietal cells located in the middle third of the gastric mucosa, and necrosis of the degenerated smooth muscle tissue within the muscular layer [16,17].

Limited information is available regarding the ultrasound characteristics of uremic gastritis in dogs. In the literature, the only relevant study is by Grooters et al., which was conducted in 1994 on four dogs with chronic uremia. In three of the included dogs, a thin hyperechoic rim was observed, outlining the mucosal surface at the interface with the gastric lumen, while in the fourth dog, the mucosa showed a moderate increase in echogenicity [18]. Both these findings were indicative of parietal mineralization [18].

This study aims to describe the ultrasonographic features of gastropathy in dogs with acute kidney injury (AKI) or acute-on-chronic kidney injury (ACKD).

## 2. Materials and Methods

### 2.1. Selection and Description of Subjects

This was a retrospective study conducted at the Veterinary Teaching Hospital of the University of Pisa from January 2014 to January 2024. Approval by Animal Research Ethics Board of University of Pisa was not required. All patients were referred to the Nephrology and Dialysis Unit of our Department for medical and/or hemodialytic management of uremia. Uremic dogs with a diagnosis of AKI or ACKD, which showed gastric wall abnormalities on abdominal ultrasound were retrospectively included. The diagnosis of AKI was based on history, clinical and hematobiochemical findings suggestive of acute impairment of renal function, and on the absence of abdominal ultrasound findings of CKD. Additionally, the diagnosis of ACKD was based on the presence of one or more of the following criteria, as previously described by Duvaneich et al.: diagnosis of CKD based on persistently increased serum creatinine concentration, with a concurrent increase in serum creatinine concentration of >25% above its previously documented baseline; abdominal ultrasound findings compatible with CKD [19]. AKI and ACKD dogs were graded according to serum creatinine, urine production, and the need for renal replacement therapies, as reported by the International Renal Interest Society (IRIS) [20].

### 2.2. Ultrasonographic Protocol

A complete abdominal ultrasound examination was conducted on the same day as the blood examination upon the patient’s admission to the Veterinary Hospital by one of 3 experienced ultrasonographers (T.P., C.P. and T.M.) with a Canon Aplio a CUS-AA000 (Canon Medical Systems Europe B.V., Zoetermeer, The Netherlands), with a 7.5 MHz microconvex probe, and/or a 12 MHz linear probe. DICOM images and video files were stored in our hospital database and retrospectively reviewed by one author (T.P.).

We included the following ultrasonographic abnormalities of the gastric wall: the presence of wall thickening, defined as greater than 5 mm [21], measured at the inter-rugal level in cases where the stomach was not distended, the layering appearance (preserved or loss of the 5-layers appearance), and changes in echogenicity affecting the mucosa and/or submucosa.

### 2.3. Statistical Analysis

Statistical analysis was carried out by one of the authors (C.P.), using commercially available statistics software (GraphPad 10.0, GraphPad Software Inc., San Diego, CA, USA). The normality was assessed by the Shapiro–Wilk test. Descriptive statistics were calculated. Means ± SD and medians with ranges were calculated depending on data distribution. Fisher’s exact test was used to evaluate potential associations between the prevalence of wall gastric alterations and the severity of AKI, and AKI vs. ACKD. The unpaired *t*-test was used to evaluate possible differences in serum calcium–phosphorus product (sCaPP) values between dogs with and without mucosal echogenicity abnormalities; moreover, the ordinary one-way ANOVA associated with Turkey’s multiple comparison test was used to investigate possible differences in sCaPP values among patients with different types of mucosal echogenicity alterations. *p*-values < 0.05 were considered statistically significant.

## 3. Results

### 3.1. Study Population

Ultrasound abnormalities of the stomach were observed in 113/343 (32.9%) of AKI and ACKD dogs which underwent abdominal ultrasound. The dogs included were 50 males and 63 females, with a median age of 5.9 years (interquartile range 2.8–9.2 years), and a median body weight of 16.9 kg (10–22.1 years). Various breeds were included, with mixed-breed dogs being the most common (n = 41), followed by Jack Russell Terriers (n = 8), Springer Spaniels (n = 7), Cocker Spaniels (n = 6), Labrador Retrievers (n = 6), German Shepherds (n = 4), Golden Retrievers (n = 3), Shar Pei (n = 3), Beagles (n = 2), Dalmatians (n = 2), Galgos (n = 2), Rottweilers (n = 2), English Setters (n = 2), Italian Spinones (n = 2), Weimaraners (n = 2), Yorkshires (n = 2), and one of the following breeds: Great Dane, American Staffordshire Terrier, Large Poodle, Border Collie, Bernese Mountain Dog, German Pointer, Hungarian Pointer, Chihuahua, Cane Corso, Coton de Tuléar, Doberman, Jagdterrier, Maltese, Australian Shepherd, Swiss Shepherd, Schnauzer, Siberian Husky, Newfoundland, and Whippet.

Among the included patients, 27 patients (23.9%) presented AKI and 86 (76.1%) ACKD, all exhibiting gastrointestinal symptoms, with anorexia/disorexia present in 100% of patients included, followed by vomiting in 63/113 (55.7%) patients and diarrhea in 17/113 (15%) of dogs.

According to the IRIS AKI grading system, the included dogs were classified as Grade II (n = 9), Grade III (n = 15), Grade IV (n = 50), and Grade V (n = 39).

### 3.2. Ultrasonographic Findings

All included patients exhibited diffuse thickening of the gastric wall. Among these, only 15 dogs (13.3%) demonstrated wall thickening without any changes in the echogenicity of the mucosa or submucosa, while 98 patients (86.7%) showed concomitant alterations in mucosal and/or submucosal echogenicity. Specifically, mucosal abnormalities were observed in 62 dogs (54.9%), submucosal abnormalities in 23 (20.3%), and combined mucosal and submucosal abnormalities in 13 (11.5%).

**Figure 1:**
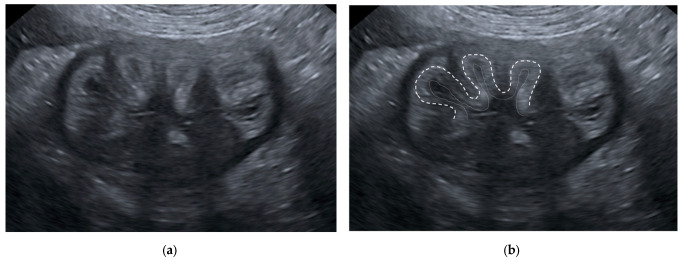
Ultrasonographic B-mode images of the stomach of a patient with a hyperechoic mucosal stripe, measuring up to 1 mm in thickness and running parallel to the mucosal surface (**a**); in image (**b**), the mucosal stripe is indicated by the dotted white line, while the solid gray line indicates the mucosal surface.

**Figure 2:**
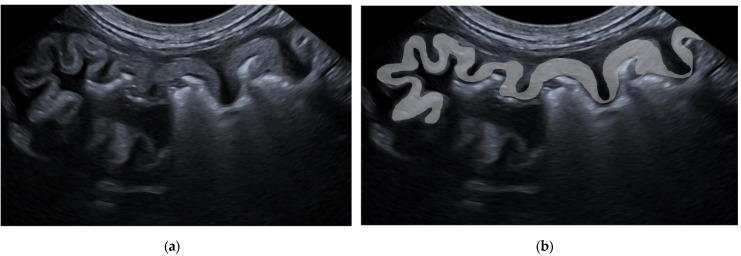
Ultrasonographic B-mode images of the stomach of a patient with a hyperechoic mucosal band with a thickness greater than 1 mm, running parallel to the mucosal surface and possibly in contact with it (**a**); in image (**b**), the mucosal band is indicated by solid gray band.

**Figure 3:**
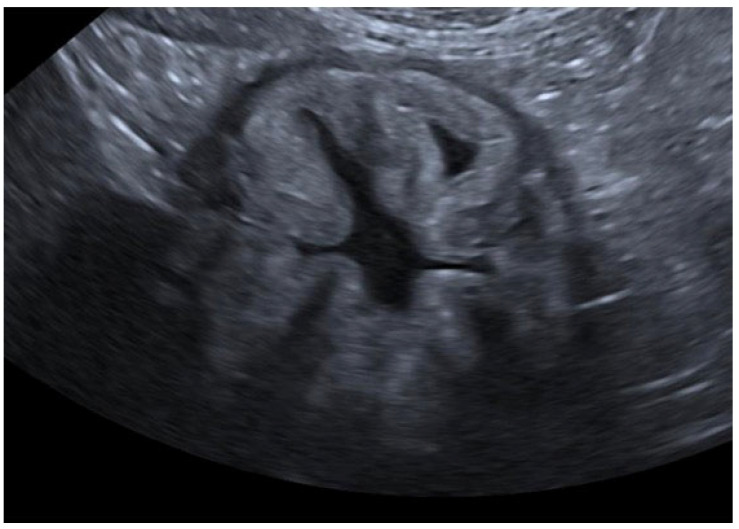
Ultrasound B-mode images of the stomach of a patient with marked diffuse mucosal hyperechogenicity.

Three distinct alterations were identified in the mucosa:

1. The presence of a hyperechoic mucosal stripe, measuring up to 1 mm in thickness and running parallel to the mucosal surface (Figure 1).
2. The presence of a hyperechoic mucosal band with a thickness greater than 1 mm, running parallel to the mucosal surface and possibly in contact with it (Figure 2).
3. The presence of marked diffuse mucosal hyperechogenicity (Figure 3).

The most prevalent finding was the mucosal hyperechoic band, observed in 48.4% of patients with exclusively mucosal alterations and 69.2% of patients with both mucosal and submucosal alterations (Table 1).

**Figure 4:**
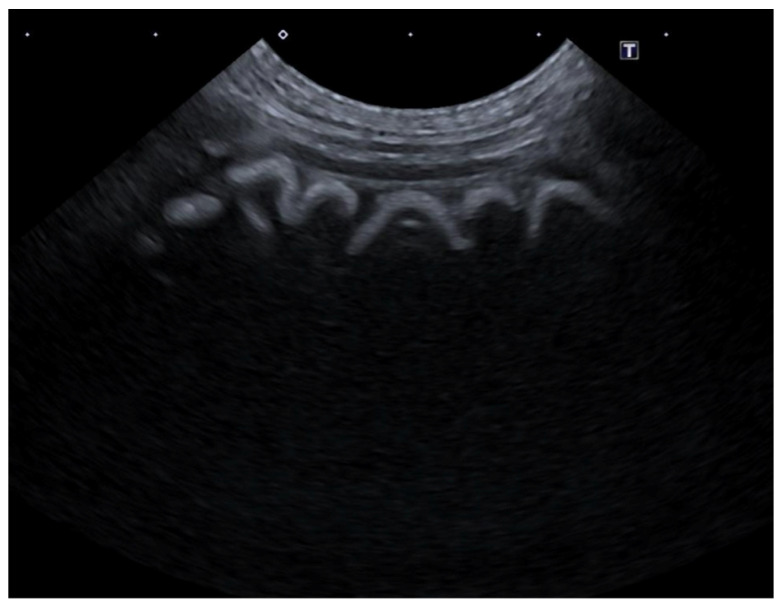
Ultrasonographic B-mode images of the stomach of a patient with posterior acoustic shadowing associated with the mucosal hyperechoic band.

In three patients exhibiting mucosal hyperechoic band, posterior acoustic shadowing was also observed (Figure 4).

**Figure 5:**
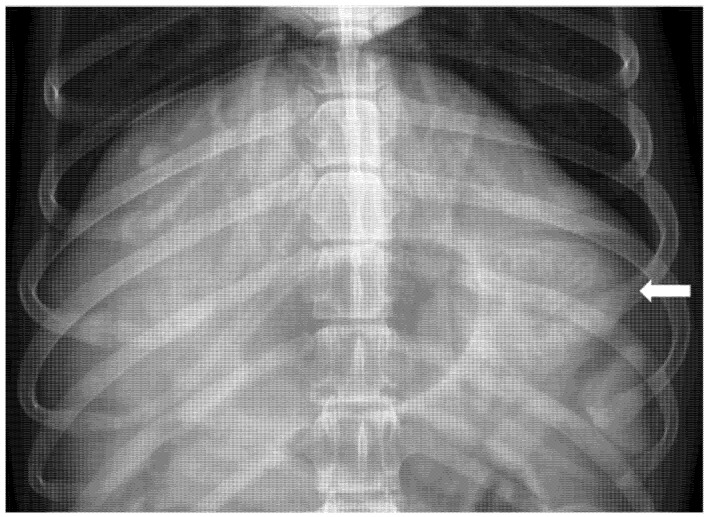
Ventro-dorsal radiographic projection of the cranial abdomen of a patient included in the study; the white arrow indicates the multiple radiopaque lines following the contour of the gastric folds, referring to gastric wall mineralization.

In 91/113 (80.5%) patients, a thoracic radiograph including the cranial abdomen was also performed; all these patients had gastric mucosal echogenicity alterations at ultrasonographic examination. Among them, 8/91 (8.8%) patients showed radiographic evidence of gastric wall mineralization, appearing as multiple radiopaque lines following the contour of the gastric folds (Figure 5).

In one patient with mucosal hyperechoic band, a post-mortem histological examination was conducted, which confirmed the mineralization of the wall.

**Figure 6:**
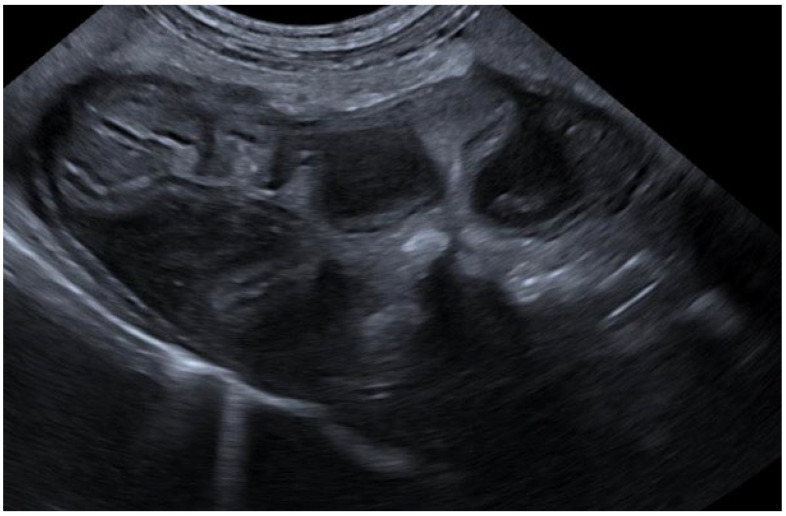
Ultrasonographic B-mode image of the stomach of a patient with thickened hypoechoic submucosa.

With regard to the submucosa, we identified alterations characterized by submucosal hypoechogenicity accompanied by a thickening of the layer. In all cases, an additional layering was observed, consisting of a centrally hypoechoic layer flanked by inner and outer hyperechoic layers (Figure 6).

Submucosal alterations were identified in 31.8% of the included patients. Among these patients, 52.7% demonstrated hypoalbuminemia in blood tests, and in three cases, pancreatic and gallbladder edema were also noted.

Considering the prevalence of gastric wall abnormalities related to the degree of AKI, we did not highlight any statistically significant differences (*p* = 0.88) among patients at different AKI grades (Table 2).

A statistically significant association was observed between the prevalence of gastric wall alterations and the presence of AKI or ACKD in the included patients (*p* < 0.0001). Specifically, exclusively mucosal alterations were more prevalent in patients with ACKD (66.3%), while exclusively submucosal alterations were more prevalent in patients with AKI (48.1%) (Table 3).

Regarding sCaPP values, a significant difference was observed between patients with mucosal alterations and those without mucosal alterations, with higher values in dogs presenting mucosal alterations (*p* = 0.013). Additionally, a significant difference was identified between patients with mucosal band and those with mucosal stripe, with higher values in the former group (*p* = 0.04). However, no significant differences were observed between patients with mucosal band and stripe or between those with mucosal stripe and diffuse mucosal hyperechogenicity. Table 4 presents the sCaPP values across the various groups analyzed.

## 4. Discussion

This study offers a comprehensive description of ultrasonographic findings of the gastric wall in dogs with AKI or ACKD. A significantly larger number of patients was considered compared to the only other ultrasound study available in the literature carried out by Grooters et al. (1994) [18]. Our results indicate a high prevalence (32.9%) of gastric wall abnormalities in patients with AKI and ACKD, underscoring the importance of gastrointestinal involvement in the course of renal dysfunction. Gastric wall thickening was observed in all patients (n = 113), most commonly associated with mucosal alterations, while submucosal alterations were less frequent.

Relatively to mucosal alterations, we highlighted findings similar to those described in the study by Grooters et al. [18]. However, our study provides a more comprehensive and detailed description of potential mucosal alterations, supported by a larger sample size. We observed the presence of diffuse mucosal hyperechogenicity or mucosal hyperechoic band/stripe, which was classified based on its thickness. Posterior acoustic shadowing was observed in some cases. The variability in mucosal changes observed may reflect different degrees of mucosal mineralization. Notably, only a small percentage of patients with mucosal alterations exhibited these changes on radiographic examination. This finding supports the hypothesis that ultrasound is more sensitive in detecting such changes in the gastric wall, as previously highlighted by Grooters et al. (1994) [18].

The study by Grooters et al. included three patients with chronic kidney disease and chronic uremia, suggesting that such alterations might develop exclusively under chronic conditions [18]. However, our findings indicate that these alterations can also occur in patients with AKI. Gastric mucosal calcification likely arises from disruptions in calcium metabolism in uremic patients [6]. As noted by Peters et al. (2005), this may represent metastatic rather than dystrophic calcification, given its association with an elevated sCaPP [4]. Similarly, cats with CKD and gastric wall mineralization exhibit a higher sCaPP compared to those without mineralization [22]. Indeed, hyperphosphatemia is a common feature of AKI, resulting from reduced renal phosphorus excretion [23]. Although hypocalcemia has been frequently observed in dogs and cats with AKI, uremic patients may also present normal to hypercalcemia [24]. Calcium–phosphate disorders can lead to an elevated sCaPP in patients with AKI or ACKD or end-stage CKD with secondary risk of metastatic calcifications [25], and, secondarily, to possible mineralization of the gastric wall. We found significantly higher sCaPP values in patients with mucosal alterations compared to those without mucosal alterations. This finding suggests that, even in dogs, there is likely an association between gastric wall mineralization and an increased sCaPP. Additionally, patients with mucosal hyperechoic band showed higher sCaPP values compared to those with a mucosal stripe and general mucosal hyperechogenicity, with significantly higher values than patients with mucosal hyperechogenicity. This finding may indicate that the mucosal band represents a greater degree of wall mineralization. This is further supported by the fact that an associated posterior acoustic shadow was observed only in association with mucosal band.

Regarding the submucosal abnormalities, our study is the first to describe such changes in patients with acute renal failure. This type of ultrasound finding could be consistent with submucosal edema [26]. Gastric wall edema has previously been reported histologically in patients with uremic gastropathy [4,18]. Ultrasonographic evidence of gastric wall edema has also been described in cases of hypoalbuminemia [26]. However, not all patients with gastric wall edema in our study had hypoalbuminemia, suggesting that its development may be associated with other factors, such as vascular abnormalities secondary to the inflammatory state caused by uremia [5,11].

A possible differential diagnosis for ultrasonographic submucosal hypoechoic thickening is submucosal hemorrhage [27,28]. Therefore, we cannot completely rule out the presence of a hemorrhagic component in the gastric wall, which could be secondary to vascular or coagulation abnormalities in the course of AKI [5,9,11]. Ultrasonographic monitoring of the stomach may help differentiate between gastric wall edema and hemorrhage, as edema typically resolves more rapidly on ultrasound than hemorrhage [29,30]. However, if both conditions coexist, or in case of persistence of edema or hemorrhage, ultrasonographic monitoring may not provide a definitive distinction.

An interesting finding of our study is that mucosal alterations were more prevalent in patients with ACKD, while submucosal alterations were more common in patients with AKI. This finding may be secondary to the fact that patients with ACKD may experience a longer duration of calcium–phosphate disorder, thus allowing more time for gastric mineralization to occur as metastatic calcifications. On the other hand, AKI patients are often oliguric, and fluid overloaded. Therefore, it is possible that the higher prevalence of submucosal alterations (including gastric edema) in the AKI group may be due to a higher risk for fluid imbalance and overload [5,9]. However, in some patients with AKI or ACKD, both alterations were observed.

This study has several limitations related to its retrospective design. First, histological examination of the gastric wall was performed in only one patient, and such analysis would have been valuable to determine whether edema or hemorrhage was present. Ultrasonographic monitoring was not conducted using a standardized protocol for all patients, which limits the assessment of the evolution of these alterations over time. Additionally, the number of animals with AKI and ACKD that did not undergo abdominal ultrasound could not be retrieved; however, in our institution, abdominal ultrasound is routinely requested by nephrology specialists as part of the diagnostic work-up. To our knowledge, no IRIS grading system has been specifically developed for patients with ACKD, yet the IRIS AKI grading system was applied to these patients in our study, which should be considered a limitation, although it is commonly used in clinical practice. Assessment of overhydration was not available for all patients due to the retrospective nature of the study, which could be relevant when interpreting sonographic findings, particularly in those with suspected gastric wall edema.

## 5. Conclusions

This study provides a comprehensive description of the ultrasonographic features of gastropathy in dogs with AKI and ACKD, highlighting the prevalence of specific mucosal and submucosal alterations. Mucosal mineralization was more commonly observed in ACKD patients, while submucosal alterations, likely related to edema, were more prevalent in AKI patients. Future research should focus on ultrasonographic monitoring of these patients to better understand the progression of these findings and their correlation with clinical signs.

## Figures and Tables

**Table 1:** Ultrasonographic mucosal abnormalities.

	Mucosal Abnormalities (n = 62)	Mucosal + Submucosal Abnormalities (n = 13)
Mucosal hyperechoic band	30/62 (48.4%)	9/13 (69.2%)
Mucosal hyperechoic stripe	16/62 (25.8%)	3/13 (23%)
Diffuse hyperechoic mucosa	16/62 (25.8%)	1/13 (7.6%)

**Table 2:** Prevalence of gastric wall abnormalities in dogs with AKI/ACKD Grade II–III and dogs with AKI/ACKD Grade IV–V; *p* = 0.88.

AKI/ACKD Grading	Mucosal Abnormalities (n = 62)	Submucosal Abnormalities (n = 23)	Mucosal + Submucosal Abnormalities (n = 13)	Exclusively Wall Thickening (n = 15)
Grade II–III (n = 24)	12/24 (50%)	6/24 (25%)	2/24 (8.3%)	4/24 (16.6%)
Grade IV–V (n = 89)	50/89 (56.2%)	17/89 (19.1%)	11/89 (12.35%)	11/89 (12.35%)

**Table 3:** Prevalence of gastric wall abnormalities in dogs with acute kidney injury (AKI) and acute-on-chronic kidney injury (ACKD); *p* < 0.0001.

	Mucosal Abnormalities (n = 62)	Submucosal Abnormalities (n = 23)	Mucosal + Submucosal Abnormalities (n = 13)	Exclusively Wall Thickening (n = 15)
AKI (n = 27)	5 (18.5%)	13 (48.1%)	5 (18.5%)	4 (7.5%)
ACKD (n = 86)	57 (66.3%)	10 (11.6%)	8 (9.3%)	11 (12.8%)

**Table 4:** Serum calcium–phosphorus product (sCaPP) expressed in mean ± standard deviation in patients with and without mucosal abnormalities and with different types of mucosal alterations; * *p* = 0.013, ** *p* = 0.04.

	sCaPP (mg/dL)
Mucosal abnormalities	156.3 ± 51.5 *
No mucosal abnormalities	128.7 ± 58.4 *
Mucosal hyperechoic band	166.1 ± 53.9 **
Mucosa hyperechoic stripe	164.6 ± 35.2
Diffuse hyperechoic mucosa	128.5 ± 52.4 **

## Data Availability

The data not presented in the manuscript are available for consultation after a reasonable request to the corresponding authors.

## Abbreviations

The following abbreviations are used in this manuscript:

AKI	acute kidney injury
ACKD	acute-on-chronic kidney injury
CKD	chronic kidney disease
IRIS	International Renal Interest Society
